# Effect of Lactic Acid Bacteria Addition on the Microbiological Safety of Pasta-Filata Types of Cheeses

**DOI:** 10.3389/fmicb.2020.612528

**Published:** 2020-12-07

**Authors:** Alžbeta Medved’ová, Martina Koňuchová, Karolína Kvočiková, Ivana Hatalová, L’ubomír Valík

**Affiliations:** Department of Nutrition and Food Quality Assessment, Faculty of Chemical and Food Technology, Slovak University of Technology in Bratislava, Bratislava, Slovakia

**Keywords:** raw milk cheeses, *Staphylococcus aureus*, *Escherichia coli*, steaming process, sensory properties, predictive microbiology

## Abstract

In this work, the effects of different combinations of lactic acid bacteria (LAB) on the growth of coagulase-positive staphylococci (CPS) and *Escherichia coli* were evaluated during ripening of 23 curd cheeses, and their subsequent behavior during the manufacture and storage of pasta-filata cheeses was characterized. Three groups of cheeses were prepared in total: first, control cheeses from raw milk without LAB addition; further pasteurized milk cheeses with LAB, CPS and *E. coli* intentional inoculation; and finally, raw milk cheeses with LAB added. The aim was to compare the effect of LAB from starter culture, and also in combination with native LAB, and to evaluate the LAB effect as a group, and further to suggest the culture with the best inhibitory potential. Based on the results, counts of CPS increased over 24 h in control curd cheese by 1.76 ± 0.56 log CFU/g. On the other hand, in raw milk cheeses with the addition of starter culture, the increase in CPS counts by 0.76 ± 0.87 log CFU/g was noticed. Counts of *E. coli* increased during the first 24 h of curd manufacture by 3.56 ± 0.41 log CFU/g in cheeses without LAB addition. Contrary to this, using of LAB cultures resulted in an increase in *E. coli* counts by only 1.40 ± 1.07 log CFU/g. After steaming at 63.6 ± 1.9°C for 7.2 ± 2.1 min (temperature of heated acidified curd was 54.9 ± 1.7°C), CPS decreased by 0.58 ± 1.12 log CFU/g, and *E. coli* decreased by 1.23 ± 0.97 log CFU/g in all cheeses, regardless of LAB addition. Finally, during storage of cheeses at 6 ± 0.5°C for 28 days, the levels of *E. coli* in control cheeses and in raw milk LAB-enriched cheeses reached levels of 2.07 ± 2.28 log CFU/g and 1.20 ± 0.85 log CFU/g, respectively. In addition, the counts of CPS at the end of storage met the criteria of EU Commission Regulation (EC) No. 1441/2007 (2007) (less than 4 log CFU/g) in all manufactured cheeses with added LAB culture, while in the control raw milk cheeses, a level of 3.80 ± 1.22 log CFU/g was observed. Regarding the culture used, the best microbiological inhibitory effect in 28-day-old cheeses was reached by the combination of Fresco culture with *Lacticaseibacillus rhamnosus* GG, and the best sensory properties were judged to be those for cheeses manufactured with Culture A. A moderate negative effect of storage on overall sensory acceptance was noted, according to the final evaluation of overall acceptability of pasta-filata cheeses. The most satisfactory overall acceptability after 28 days of storage at 6°C was reached for cheese with the addition of culture A.

## Introduction

The pasta-filata cheeses include a wide range of cheeses originating primarily in the northern Mediterranean region: e.g., Italy, Greece, the Balkans, and Turkey. They have become global favorites as ingredients in a variety of foods, especially pizza (Albenzio et al., 2013). Some of these types of cheeses are artisanal variants that are also produced in Central and Southeast Europe. In Slovakia, the manufacture of pasta-filata type cheeses: e.g., Parenica, Oštiepok, Zázrivské vojky, Zázrivský korbáčik, and Oravský korbáčik, is of great importance to preserve national gastronomic heritage, and they are designated for the Protections of Geographical Indications (PGI) (2005) (EC No: SK/PGI/005/0485/19.07.2005). Traditionally, they are produced from lump cheese, which is also manufactured from raw milk in Slovakian upland cottages immediately after milking. The lump cheese is curdled with rennet, fermented by native lactic acid bacteria (LAB), and briefly ripened for 24 h. Then, the lumps are steamed at 60–70°C in hot water for 5–10 min, cooled (usually in salty water) and shaped (Licitra et al., 2018).

Artisanal pasta-filata cheeses are produced by a stretching process occurring after dipping a curd in hot water. During this process, the amorphous protein structure of the curd is transformed into an oriented structure composed of parallel protein fibers. The ongoing changes are influenced by an appropriate combination of *pH* level and calcium content in the curd during the heating-stretching step that is controlled by the activity of the starter cultures or by a direct acidification of milk with organic acids during the production of the curd (Zimanová et al., 2016). The resulting plastic paste is easily molded into various forms and shapes (Albenzio et al., 2013).

From the microbiological point of view, microorganisms, including LAB, live in clusters incorporated into the casein network and remain mostly in the curd, while some microorganisms leave the curd with whey (McSweeney, 2004). The development of curd fermentation relies on the spontaneous development of LAB, which produce lactic acid, helping the milk coagulate (Wouters et al., 2002). Stretching the cheese curd in hot water substantially affects the subsequent distribution and viability of the microbiota present; however, it does not destroy it completely (Gernigon et al., 2010; Hui et al., 2012). This is why residual lactose is further metabolized by bacterial survivors during pasta-filata manufacture and early stages of shelf life. Caseins that were hydrolyzed initially through residual coagulant activity by plasmin and other indigenous proteolytic enzymes to a range of large and intermediate peptides are further hydrolyzed by LAB proteinases and peptidases to shorter peptides and amino acids. As a consequence of hydrolysis and changes in water-binding ability, including changes in *pH* that in turn may cause other changes such as the migration and precipitation of calcium phosphate, pasta-filata cheese texture may soften (McSweeney, 2004). Thus, the flavor of pasta-filata cheese is very mild; other flavor notes are readily detected at low intensities and are generally considered defects (Kindstedt et al., 2010).

Despite a short fermentation of lump cheese, the metabolic activity of LAB leads to the desired degradation of saccharides, lipids, proteins and other milk components (e.g., citrates, inorganic compounds), forming a wide range of metabolites that improve the technological and sensory properties of the final product (Marth and Steele, 2001; Ljungh and Wadström, 2009; Lahtinen et al., 2011; Mančušková et al., 2018). In addition to organic acids (lactic, acetic, benzoic, formic, pyroglutamic, phenyllactic), other substances (e.g., hydroperoxide, diacetyl, acetoin, exopolysaccharides, etc.) that contribute to the flavor, aroma and textural properties of dairy products are produced by the action of LAB. The metabolism of proteins results in the production of various amino acids that are precursors for aldehydes, alcohols, carboxylic acids, hydroxyacids, dimethyl sulfide, dimethyl disulfide or dimethyl trisulfide and methanethiol (Østlie et al., 2003; McSweeney, 2004; Sádecká et al., 2014; Kowalczyk et al., 2016). Through the activity of lipases and esterases various free fatty acids, methyl ketones, lactones, thioesters, and ketoacids are formed (Pot and Tsakalidou, 2009). These metabolic products serve as a tool for controlling the growth and multiplication of spoilage and pathogenic microorganisms synergistically with a lowering of the *pH* (Adams, 2001; Wouters et al., 2002; Charlier et al., 2008). Moreover, in addition to the inhibitory effect of LAB, they are widely used because of their positive effects on the sensory properties of final products. However, it should be kept in mind that thermal treatment influences not only the microbiological, biochemical, physicochemical, and functional characteristics of the cheese but also, in turn, markedly affects the sensory properties of these products (Kindstedt et al., 2010). Therefore, the choice of an appropriate active starter culture for controlled fermentation processes and production of pasta-filata cheeses with thermal treatment is of utmost importance.

Despite all the benefits of cheese consumption and even though cheese is considered one of the safest foods, pathogenic bacteria can be transmitted by dairy products since their presence in raw milk cannot be completely avoided. Moreover, microbial contamination can impart a considerable load of microorganisms during the steaming process and ultimately determines the microbiological quality and safety of the final pasta-filata cheeses. Originating from raw milk or from manufacturing environments, salmonellae and *Listeria monocytogenes* are rare, and *Escherichia coli* and *Staphylococcus aureus* are more frequent microbial contaminants of lump cheeses (Little et al., 2008; Valík, 2013). The prevalence of *Listeria monocytogenes*, *E. coli* and coagulase-positive staphylococci in raw milk Slovak cheeses was found to range from 0.96 – 5.26%, 45.21 – 86.96%, and 9.96 – 12.28%, respectively from 2015–2018 (Ministry of Agricultural and Rural Development of the Slovak Republic, 2020).

Generally, it is known that efficient inhibition of microbial contaminants requires fast growth and high metabolic activity of LAB. As steaming is the only process responsible for inactivating undesirable microbiota, the following questions associated with the roles of LAB remain to be answered in this study. First, to what extent can LAB control the growth of accompanying microorganisms before steaming? The question of what proportions of high LAB numbers survive the steaming process naturally follows. On the other hand, it is also necessary to know the heat resistance of relevant representative microbiota, including LAB. Additionally, can LAB survivors control the behavior of other survivors? If yes, for what a period of time they do not affect sensorial properties of final pasta-filata cheeses? To answer these questions, we performed quantitative experiments aimed at the addition of various LAB cultures to increase the overall quality of specific traditional pasta-filata cheeses.

## Materials and Methods

### Microorganisms and Preparation of Microbial Suspensions

During cheese manufacture, several microbial cultures were used:

– Fresco DVS 1010 (Christian Hansen, Hørsholm, Denmark) consisting of *Lactococcus* (*L.*) *lactis* ssp. *lactis*, *L. lactis* ssp. *cremoris*, and *Streptococcus salivarius* ssp. *thermophilus*;
– Culture A (Rajo, Inc., Bratislava, Slovakia) consisting of *L. lactis* ssp. *lactis*, *L. lactis* ssp. *cremoris*, *Leuconostoc mesenteroides* ssp. *cremoris*, and *Lactobacillus* (*Lb.*) *acidophilus*;
– *Lb. acidophilus* LA145 (Christian Hansen, Hørsholm, Denmark);
– *Lacticaseibacillus* (*Lcb.*) *rhamnosus* VT1 isolated from tartar sauce by Assoc. Prof. Plocková at the University of Chemistry and Technology in Prague (Czech Republic);
– *Lcb. rhamnosus* GG provided by Dr. Salminen (University of Turku, Turku, Finland)

A few grains of the frozen Fresco culture were inoculated into 100 ml of sterile milk and incubated at 30 ± 0.5°C for 5 h until the stationary phase was reached. After that, 40 ml of this culture (in the case of PM7 cheese 150 ml of culture was used) was then inoculated into milk to gain a density of 5 log counts. Culture A was inoculated into the milk used for cheese manufacture, directly from commercial packaging, to reach a cell counts of 5 log CFU/g. *Lcb. rhamnosus* GG, VT1 and *Lb. acidophilus* LA 145 were inoculated into 10 ml of sterile MRS broth (Biokar Diagnostics, Beauvais, France), and incubated anaerobically (5% CO_2_) at 37 ± 0.5°C for 48 h. After that, 30 ml of each culture was inoculated into milk to obtain densities of 6 log counts.

For the cheeses manufactured from pasteurized milk, *Staphylococcus aureus* and *Escherichia coli* strains and isolates were used to analyze the effect of LAB on their growth ability or survival during cheese manufacture. The strains used were:

– *S. aureus* CCM 3953 and *E. coli* CCM 3988 from the Czech Collection of Microorganisms.

In addition, certain *S. aureus* and *E. coli* isolates were used. All these isolates are maintained in a collection of the Department of Nutrition and Food Quality Assessment, Slovak University of Technology in Bratislava, where we positively identified them by standard microbiological, biochemical and molecular methods and by MALDI-TOF spectroscopy.

– *S. aureus* 16 isolated from pasta-filata cheese “Parenica,”
– *S. aureus* 14733 isolated from a vending machine drain valve, producer of SED,
– *S. aureus* 2064 isolated from ewes’ lump cheese,
– *S. aureus* 9V1 isolated from a laboratory-produced pasta-filata cheese from raw cows’ milk,
– *E. coli* BR isolated from ewes’ cheese “Bryndza,”
– *E. coli* KF isolated from raw cows’ milk,
– *E. coli* PSII isolated from laboratory-produced pasta-filata cheese from raw cows’ milk,
– *E. coli* ZV isolated from ewes’ lump cheese.

A standard suspension of each isolate was prepared from a 24-h-old culture grown in BHI broth (Sigma-Aldrich, St. Louis, Missouri, United States) at 37 ± 0.5°C. These cultures were then diluted in saline-peptone solution and from 10^–3^ dilutions; 0.6 ml of each culture was inoculated into milk to obtain densities of 3 log counts.

### Cheese Manufacture and Microbiological Analysis

**FIGURE 1:**
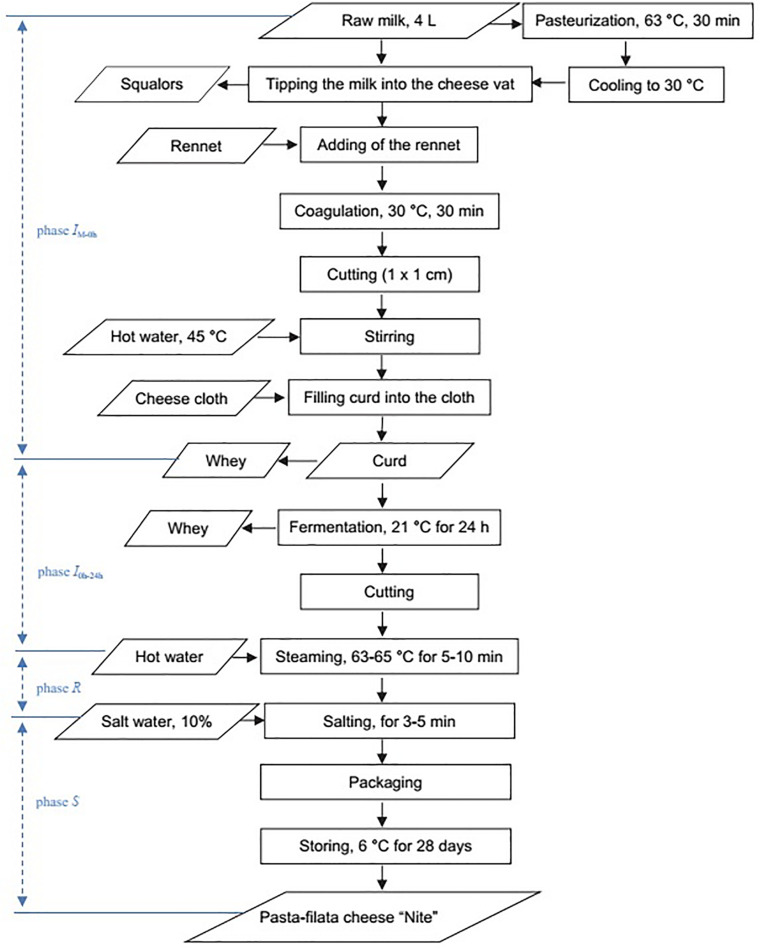
Flow diagram of production of pasta-filata “Nite” cheeses.

The raw cows’ milk was obtained between November 2018 and March 2020 from a milk vending machine situated in Bratislava, Slovakia, and immediately transported into the laboratory in cooled boxes at 6 ± 0.5°C. Manufacture of the pasta-filata “Nite” cheeses followed the scheme depicted in Figure 1 and Supplementary Material. The rennet used for cheese manufacture was Fromase (220 IMCU/ml; DSM, Heerlen, the Netherlands).

The raw milk, whey, fresh curd, lump cheese (at 3, 6, and 24 h), fresh pasta-filata cheese, pasta-filata cheese after 7, 10, 14, 21, and 28 days of storage, salty water and hot water were analyzed to enumerate LAB (presumptive counts of lactococci and presumptive counts of lactobacilli, hereafter denoted only as counts of lactococci or lactobacilli), total coagulase-positive staphylococci (CPS) and *E. coli*.

The actual counts of lactococci were determined on M17 agar (Biolife, Milan, Italy) according to EN ISO 15214 (2002), with aerobic incubation at 30 ± 0.5°C for 48 h. Numbers of lactobacilli were determined according to EN ISO 15214 (2002) on MRS agar (Biokar Diagnostics, Beauvais, France), with anaerobic (5% CO_2_) incubation at 37 ± 0.5°C for 72 h. Counts of *E. coli* were determined according to National Standard Method F23 (2005), with incubation at 37 ± 0.5°C for 24 h. Presumptive counts of CPS were determined on Baird-Parker medium (Merck KGaA, Darmstadt, Germany) with aerobic incubation at 37 ± 0.5°C for 48 h, according to EN ISO 6888-1 (2001).

The actual temperatures and duration of each steaming process were recorded by a temperature data logger (Ebro EBI 100-T100 with internal sensor, −40 to + 150°C; Xylem Analytics Germany Sales GmbH & Co., KG Ebro, Ingolstadt, Germany) connected to Winlog. pro 2.66 software, 2014 (Xylem Analytics Germany Sales GmbH & Co., KG Ebro, Ingolstadt, Germany). Moreover, for the same samples, water activity (*a*_*w*_) measured by a LabMaster-aw (Novasina, Lachen, Switzerland) and *pH* values measured with a 100L pH meter (VWR International GmbH, Wien, Austria) equipped by glass electrode Sen Tix 81 (WTW GmbH, Weilheim, Germany) were also determined.

### Sensorial Analysis of Cheese

#### Sensory Scoring of Cheeses

Quantitative descriptive analysis (QDA), based on a procedure described by Stone et al. (2012), was used to quantify the sensory attributes of the produced pasta-filata cheeses. The detailed sensory profile procedure was performed according to ISO 13299 (2016) in the Laboratory of Sensory Analysis of the Slovak University of Technology in Bratislava by a panel composed of 10 trained assessors (3 male, 7 female, aged 20–60, ISO 8586, 2012). The sensory evaluation was performed using a sensory profile of fresh and stored (6 ± 0.5°C for 10, 14, and 28 days) pasta-filata cheeses. Six samples were submitted for this evaluation (CC1, RM1, RM 4, RM6, RM8, and RM9). None of PM cheeses was evaluated due to the presence of enterotoxinogenic isolate *S. aureus* 14733. Approximately 20 g of each cheese sample was placed at ambient temperature (approximately 20°C) on a plastic plate 30 min before sampling and coded with a random 3-digit number. Overall sensory quality using a 5-point hedonic scale for each attribute was evaluated (1 and 5 representing “absence of sensation” and “highest intensity of observable sensory parameter,” respectively). Each cheese sample was evaluated for appearance (color intensity, color homogeneity/uniformity and visual suitability), texture (chewiness, springiness/elasticity, stickiness), aroma (milky, acidic, intense, rancid, moldy, cowshed, grassy, spicy, fruity) and taste (bitter, rotten, oxidized/metallic, cowshed, grassy, spicy, fruity, taste of fresh cheese, milky, acidic, acidic taste suitability). After tasting, each panelist rated the overall acceptability of the product. Assessors used plain crackers and water to clean their palates in between tasting of samples.

### Statistical Analysis

Statistical analysis of the results obtained was performed using Microsoft Excel 365 (Microsoft, Redmond, United States). The statistical significance of differences in the mean counts of microorganisms and in sensory parameters in different cheeses was evaluated by using ANOVA model. When differences were significant (*p* < 0.05), the means were compared with Tukey’s *post-hoc* test.

## Results

### Production of Lump Cheeses

“Nite” cheeses are typical Slovak pasta-filata cheeses of thick (2–10 mm) string shape. They are very popular in Slovakia, being sold in supermarkets, shops with traditional food products, markets and even in refrigerated vending machines. These products are usually consumed as snacks (Tomáška et al., 2019). To study the effect of LAB presence on microbiological and sensory quality of pasta-filata cheeses, we prepared 23 cheeses divided into 3 groups as specified in Table 1: 5 cheeses from raw milk without any added LAB culture (denoted CC group – control cheeses), 8 cheeses made from pasteurized milk with intentional inoculation of *S. aureus* and/or *E. coli* and LAB culture (denoted PM group) and finally, 10 cheeses from raw milk with addition of different LAB cultures (denoted RM group).

**TABLE 1:** Characterization of manufactured pasta-filata cheeses, depending on the addition and intended inoculum concentration of starter, additional cultures of LAB, or intentional inoculation of *S. aureus* and *E. coli* mixture.

**Group**	**Cheese**	**Starter culture with intended final concentration**	**Additional culture with intended final concentration**	**Inoculated undesirable MO with intended final concentration**
CC	CC1 – CC5	None	None	None
PM	PM1	Fresco 10^6^ CFU/ml	None	*E. coli* mixture 10^3^ CFU/ml
	PM2	Fresco 10^6^ CFU/ml	None	*S. aureus* mixture 10^3^ CFU/ml
	PM3	Fresco 10^6^ CFU/ml	None	*E. coli* mixture + *S. aureus* mixture twice, 10^3^ CFU/ml
	PM4	Culture A 10^6^ CFU/ml	None	*E. coli* mixture 10^3^ CFU/ml
	PM5	Culture A 10^6^ CFU/ml	None	*S. aureus* mixture 10^3^ CFU/ml
	PM6	Culture A 10^6^ CFU/ml	None	*E. coli* mixture + *S. aureus* mixture 10^3^ CFU/ml
	PM7	Fresco 10^7^ CFU/ml	None	*E. coli* mixture + *S. aureus* mixture 10^3^ CFU/ml
	PM8	Culture A 10^7^ CFU/ml	None	*E. coli* mixture + *S. aureus* mixture 10^3^ CFU/ml
RM	RM1 - RM3	Fresco 10^6^ CFU/ml	None	None
	RM4, RM5	culture A 10^6^ CFU/ml	None	None
	RM6	Fresco 10^6^ CFU/ml	LA145 10^6^ CFU/ml	None
	RM7	Fresco + culture A 10^6^ CFU/ml	None	None
	RM8	Fresco 10^6^ CFU/ml	VT1 10^6^ CFU/ml	None
	RM9	Fresco 10^6^ CFU/ml	LGG 10^6^ CFU/ml	None
	RM10	Fresco (ewes’ milk) 10^6^ CFU/ml	LGG 10^6^ CFU/ml	None

*CC – control cheeses without the addition of LAB, PM – cheeses from pasteurized milk with the addition of culture A or Fresco and intentional inoculation with a mixture of 5 isolates of *S. aureus* and/or *E. coli*, RM – raw milk cheeses with the addition of different LAB cultures, LA145 – *Lb. acidophilus* LA145, VT1 – *Lcb. rhamnosus* VT1, GG – *Lcb. rhamnosus* GG.*

Applying the rationale of the Food Safety Objective concept (International Commission for the Microbiological Specification of Foods (ICMSF), 2002), the initial counts *H*_0_ and the steps associated with the increase (*I*) or reduction (*R*) of microbial counts were identified to assess their total increase Σ*I* or decrease Σ*R*. As all the procedures were performed in the laboratory and mostly using close-to-sterile utensils, the increase in microbial numbers from possible recontamination was not taken into consideration. The average initial microbial numbers (*H*_0_) among all milk samples varied as follows: 4.55 ± 0.98 log CFU/ml for lactococci, 3.82 ± 0.80 log CFU/ml for lactobacilli, 1.86 ± 1.03 log CFU/ml for *E. coli* and 2.94 ± 0.51 log CFU/ml for CPS.

### Milk Coagulation

**FIGURE 2:**
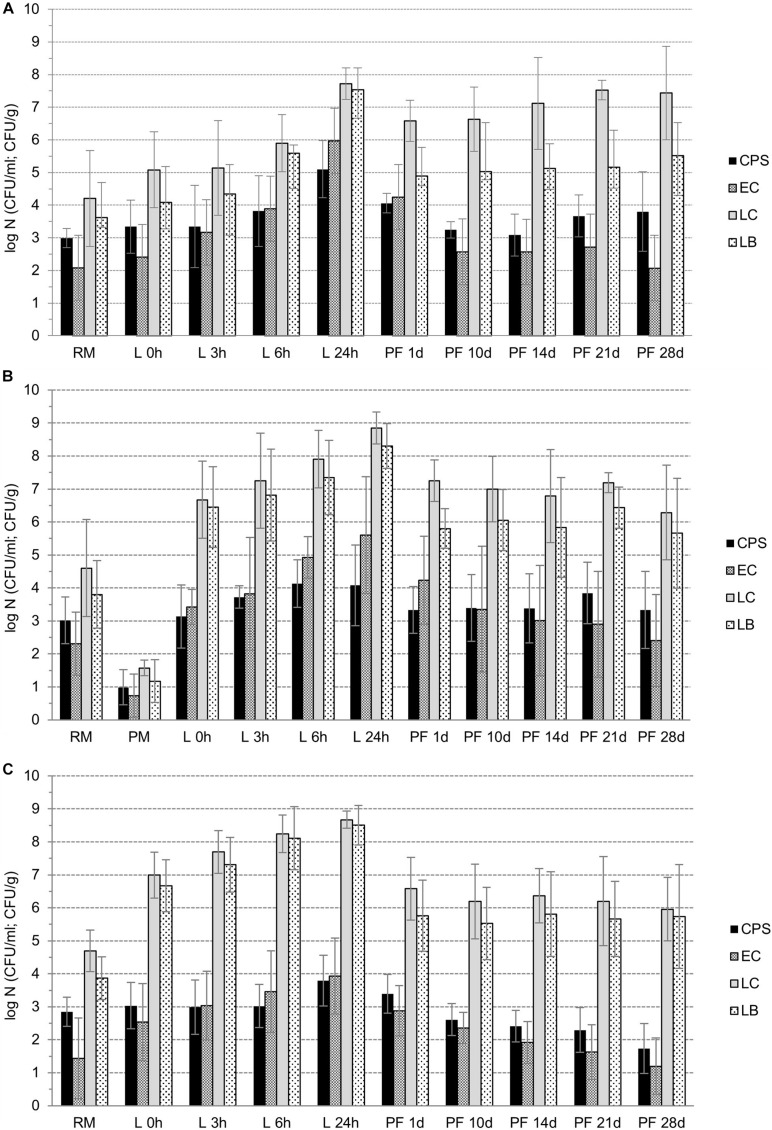
**A–C**: The counts of selected microbial groups (CPS – coagulase-positive staphylococci, EC – *E. coli*, P-LC – presumptive counts of lactococci, P-LB – presumptive counts of lactobacilli) during manufacture of pasta-filata cheeses from raw milk (RM) to curd (L 0h), during fermentation of curd (L 3 h, L 6 h, and L 24 h), and during manufacture and storage of pasta-filata cheeses (PF1d – PF28d) at 6 ± 0.5°C for 28 days dependent of various cheese groups (**A** – CC **B** – PM, **C** – RM).

**FIGURE 3:**
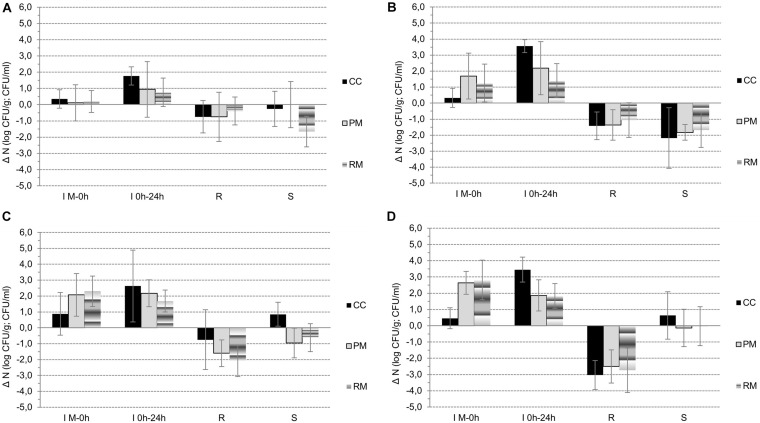
**A–D**: The changes in microbial counts (**A** – CPS, **B** – *E. coli*, **C** – lactococci, **D** - lactobacilli) during the manufacture of curds from raw milk to curd (phase G1), during fermentation of curd (phase G2), and steaming (phase D) and storage (phase S) of CC, PM and RM pasta-filata cheeses.

Naturally, during the manufacture of cheeses from raw milk (M) until the fresh curd (0h) preparation (phase *I*_*M–*__0__*h*_, Figures 2A–D, 3A–C), the most statistically significant (*p* < 0.05) increases in lactobacilli (*I*_*G*__1_,_*LB*_ = 2.73 ± 1.00 log CFU/g) and lactococci (*I*_*M–*__0__*h*_,_*LC*_ = 2.20 ± 1.11 log CFU/g) were observed in curds with LAB culture addition, in both the PM and RM groups. However, in CC curds *I*_*M–*__0__*h*_,_*LB*_ was only 0.46 ± 0.65 log CFU/g and *I*_*M–*__0__*h*_,_*LC*_ = 0.88 ± 1.34 log CFU/g. As a result of intentional inoculation of *E. coli* its growth was more intensive in PM group (*I*_*M–*__0__*h*__,EC_ = 1.69 ± 1.43 log CFU/g) compared to both the CC and RM groups, with *I*_*M–*__0__*h*_,_*EC*_ = 0.94 ± 1.10 log CFU/g. In the case of CPS, their growth during curd preparation was only minimal, and the highest increment of *I*_*M–*__0__*h*__,EC_ = 0.35 ± 0.57 log CFU/g was noticed in the CC group.

### Distribution of Microorganisms Between Curds and Whey

**FIGURE 4:**
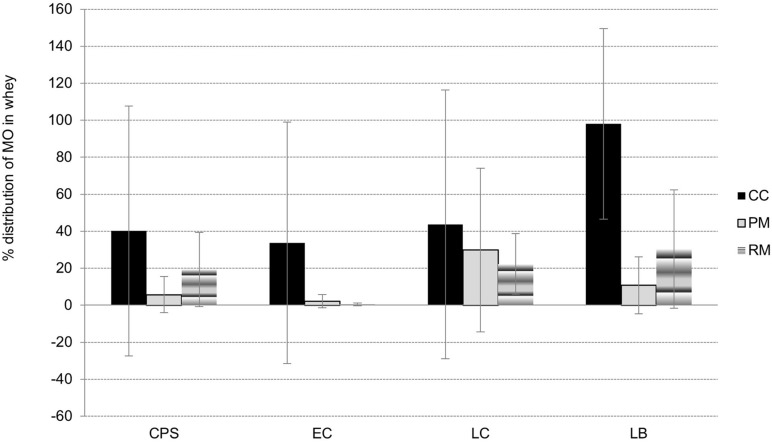
The distribution of microbial groups (CPS – coagulase-positive staphylococci, EC – *E. coli*, LC – presumptive counts of lactococci, LB – presumptive counts of lactobacilli) between whey and curd during manufacture of lump cheeses for CC, PM, and RM, later used as raw materials for pasta-filata cheeses.

As a consequence of syneresis, microbial partitioning between the curds and whey occurred. As one shown in Figure 4, where the partitioning {*%W/L* = (*N_*whey*/_N_*L* 0*h*_*) ^∗^ 100%} during syneresis is depicted, the presence of all microbial groups in whey was lower for LAB-enriched cheeses. This was especially true in the case of *E. coli*; its ratios in whey from cheeses with LAB culture addition (group PM and RM) were only 2.1 ± 3.6% and 0.4 ± 0.8%, respectively. Additionally, most CPS remained in curd (94.3 ± 9.8% and 80.7 ± 20.0%) in both PM cheeses and RM cheeses, respectively, thus indicating the need for their rapid inhibition during further cheese fermentation. On the other hand, as the initial counts of LAB in milk were higher (as a result of their addition), and they grew faster, inhibitory activity against *E. coli* and CPS was achieved earlier.

### Fermentation of Curds

During the next 24-h lasting fermentation step (phase *I*_0*h–*24*h*_, Figures 3A–D), a further increase in microbial counts (*I*_0*h–*24*h*_) was naturally observed. In all microbial groups, the largest *I*_0*h–*24*h*_ was noted for CC curds, with a declining tendency occurring gradually in PM and RM curds. At the same time and in all the curds, values of *I*_0*h–*24*h*_,_*EC*_ > *I*_0*h–*24*h*_,_*CPS*_, and, specifically *I*_0*h–*24*h*_,_*EC*_ was 3.56 ± 0.41 log CFU/g, 2.18 ± 1.66 log CFU/g and 1.40 ± 1.07 log CFU/g in CC, PM and RM curds, respectively. For CPS in the same order of curds, values of *I*_0*h–*24*h*_,_*CPS*_ were only 1.76 ± 0.56 log CFU/g, 0.94 ± 1.71 log CFU/g and 0.76 ± 0.87 log CFU/g, respectively. Finally, similar declining trends of *I*_0*h–*24*h*_,_*LC*_ and *I*_0*h–*24*h*_,_*LB*_ were found for CC, PM and RM curds, respectively.

Despite the growth of CPS and *E. coli* during fermentation (Figures 3A–D), the addition of LAB cultures finally led to their statistically significant (*p* < 0.05) suppression in PM and RM compared to CC curds. Moreover, there was also a higher total increase (*I*_*M–*0*h*_ + *I*_0*h–*24*h*_ phases) of LAB in cheeses with LAB addition. As a result of LAB activity, the counts of CPS and *E. coli* after 24 h of fermentation were lower than 4 log CFU/g in all 18 LAB-enriched cheeses. In contrast, the average counts of CPS and *E. coli* reached values of 5.10 ± 0.87 log CFU/g and 5.97 ± 0.55 log CFU/g in the CC group, respectively.

### Steaming Process

The initial *pH* value of the fresh curds was 6.4 ± 0.2. Curds suitable for steaming should have a *pH* of approximately 5.0–5.3 (Fuentes et al., 2015; Pappa et al., 2019), as a lower *pH* results in a soft curd that collapses during stretching (Niro et al., 2014). In laboratory-manufactured cheeses, after 24 h of fermentation, the *pH* values of curds in the CC group decreased to *pH* = 6.0 ± 0.5. In PM and RM cheeses, significantly lower (*p* < 0.05) *pH* values were noticed after 24 h of fermentation, specifically, *pH* = 5.2 ± 0.3 in the PM cheeses group and *pH* = 5.1 ± 0.1 in the RM cheeses group. All the curds were cooked for 7.2 ± 2.1 min in hot water at 63.6 ± 1.9°C, resulting in a curd temperature of 54.9 ± 1.7°C recorded by the Ebro EBI 100-T100 temperature data logger.

The observed reduction (*R*) of all microbial groups as a result of the steaming process is depicted in Figures 2A–C and in Figures 3A–D. Comparing the viability of lactococci and lactobacilli, the former ones exhibited higher tolerance to the heating process. The values of *R*_*LB*_ ranged from 2.50 ± 1.02 log CFU/g in the PM cheese group to 3.03 ± 0.89 log CFU/g in the CC cheese group. The *R*_*LC*_ values were lower; 0.75 ± 1.88 log CFU/g, 1.60 ± 0.84 log CFU/g and 2.09 ± 0.98 log CFU/g in CC, PM and RM, respectively. While lactococci survivors in fresh pasta-filata cheeses remained above 6.5 log (6.58 ± 0.79 log CFU/g, 7.25 ± 0.63 log CFU/g and 6.58 ± 0.95 log CFU/g), counts of lactobacilli only exceeded 4.5 log counts (4.89 ± 0.88 log CFU/g, 5.80 ± 0.60 log CFU/g and 5.79 ± 1.07 log CFU/g) in CC, PM and RM cheeses, respectively.

Although the reduction of undesirable microbiota was lower in LAB-enriched cheeses, it seems that this may have resulted from their previous slower growth during phase G. The decrements of *E. coli* counts (*R*_*EC*_) were calculated to be 1.42 ± 0.86 log CFU/g, 1.37 ± 0.95 log CFU/g and 1.06 ± 1.08 log CFU/g in CC, PM and RM, respectively. CPS proved to be more thermotolerant, as their *R*_*CPS*_ values were only 0.75 ± 1.00 log CFU/g, 0.75 ± 1.51 log CFU/g and 0.40 ± 0.86 log CFU/g, respectively, for the same order of cheese groups. However, it is also important to note that both *E. coli* and CPS surviving populations in the CC group of cheeses without LAB addition were higher than 4 log counts, while the counts for CPS in PM and RM cheeses were 3.33 ± 0.71 log CFU/g and 3.40 ± 0.59 log CFU/g, respectively. Numbers of *E. coli* lower than 3 log CFU/g after steaming were surprisingly observed only in fresh RM pasta-filata cheeses with LAB added (2.88 ± 0.76 log CFU/g).

### Refrigerated Storage

To determine further quantitative changes in selected microbial populations, the fresh pasta-filata cheeses were stored at 6 ± 0.5°C for 28 days and analyzed regularly after 10, 14, 21, and 28 days. The results are depicted in Figures 2A–C and in Figures 3A–D (storage phase S, during that increase in microbial counts – labeled as *I*s; or decrease of their counts – labeled as *R*_*S*_ was observed). Only in control raw milk pasta-filata cheeses did lactococci and lactobacilli grow during storage, represented by *I*s,_*LC*_ = 0.85 ± 0.75 log CFU/g and *I*s,_*LB*_ = 0.63 ± 1.45 log CFU/g. In other cheeses with LAB addition, the lactobacilli remained at the same level after steaming, lactococci numbers decreased on average less than 1 log CFU/g, *R*_*S*,*LC*_ = 0.96 ± 0.93 CFU/g in the PM group and *R*_*S,LC*_ = 0.63 ± 0.88 CFU/g in the RM group. At the end of storage the counts of lactococci in the CC, PM and RM groups of cheeses were 7.43 ± 0.43 log CFU/g, 6.28 ± 1.43 log CFU/g and 5.96 ± 0.96 log CFU/g, respectively, significantly (*p* < 0.05) lower than those in the CC group. On the other hand, the counts of lactobacilli in all cheese groups on day 28 were higher than 5.5 log CFU/g, with no statistically significant (*p* < 0.05) differences between groups.

Higher levels of LAB together with their inhibitory metabolites acted as the hurdle for other microbial contaminants. This was confirmed in all groups of cheeses, as further reductions (*R*_*S*_) were observed in both CPS and *E. coli* during storage of pasta-filata cheeses despite the fact that differences between the counts in each group of cheeses were not statistically significant (*p* < 0.05). For example, for *E. coli*, the final reductions *R*_*S*,*EC*_ were approximately 2 log CFU/g in 28 days of storage. The lowest final *E. coli* counts of 1.20 ± 0.85 log CFU/g were reached in RM pasta-filata cheeses, while in the CC and PM groups, the *E. coli* counts exceeded 2 log CFU/g. For CPS, there was a statistically significant decrease (*p* < 0.05) during storage of RM pasta-filata cheeses (*R*_*S*,*CPS*_ = 1.5 log CFU/g) and a difference in final counts of CPS between PM and RM cheeses. The most important results is the observation that the final average counts of CPS were lower than 4 log CFU/g in all groups of cheeses, specifically 3.80 ± 1.22 log CFU/g, 3.33 ± 1.17 log CFU/g and 1.73 ± 0.76 log CFU/g in RM, PM and RM pasta-filata cheeses, respectively.

### Effect of LAB on the Sensory Quality of Pasta-filata Cheeses

The panelists did not determine significant differences (*p* < 0.05) in appearance between the LAB-enriched cheeses and the CC cheeses and described them as being white to creamy white, with the overall uniformity in color that is a basic requirement of pasta-filata cheeses (Council Regulation (EC) No 510/2006, 2006; Zimanová et al., 2016). However, color intensity and uniformity tended to decrease between the 1st and 28th days (4.6 ± 0.6 to 4.2 ± 0.8 and 4.9 ± 0.3 to 4.5 ± 0.7, respectively). The most stable scoring of both parameters was noticed for RM9 cheese, and its rating did not decrease below 4.5 points even at the end of the consumption period. Fresh pasta-filata cheeses exhibited visual suitability ranging from 4.1 to 4.9 points (on average 4.6 ± 0.3), which indicates a very acceptable appearance for consumers. The storage influenced the sensory evaluation of this parameter, which it decreased approximately 0.8 points after 28 days of storage.

### Mechanical Texture

Mechanical texture attributes such as chewiness, elasticity and stickiness did not significantly (*p* < 0.05) change among samples during the study. The mean values of those characteristics were 4.0 ± 0.4, 3.8 ± 0.6, and 4.5 ± 0.3 points, respectively, on the 5-point scale used. In general, not too much force was required to masticate to a state ready for swallowing, and cheeses were able to regain their initial thickness rapidly after compression and deformation. Nevertheless, the panelists perceived better textural properties in RM cheeses in comparison with products without the LAB addition, especially in the case of chewiness (4.2 ± 0.8 vs. 3.1 ± 0.9). However, the sensory profiles reported by the assessors’ panel showed no noteworthy differences.

### Aroma and Taste

The indicators of aroma and taste were classified by the descriptors that could have positive or negative effects on the overall sensory value of pasta-filata cheeses. In final products, the main purpose was preservation of primary descriptors - delicious milky, slightly sour and mild in flavor. On the other hand, the evaluation of possible negative effects (rancid, moldy, cowshed or other foreign) on the overall flavor was an important step in the assessment.

The panelists perceived higher intensities of milky aroma (*p* < 0.05) in RM cheeses (3.6 ± 1.0) than in CC cheeses (2.7 ± 0.8). Furthermore, the addition of selected LAB adjuncts positively controlled the aroma intensity. RM cheeses received hedonic scores near the neutral point (3.4 ± 0.8), indicating appropriate mild aroma of the prepared products. Control sample CC1 featured a higher aroma intensity (4.4 ± 0.7). The intensity of acidic aroma is a descriptor that can increase the sensory acceptance and quality of pasta-filata cheeses. Scores for acidic aroma above 3.0 could be considered unpleasant and disturbing. The results of this attribute were not affected by the adjunct cultures used. The panelists rated the acidic aroma of all the cheese samples as weak to moderate (from 2.0 to 3.0), which corresponds to an average value of 2.4 ± 0.3. The storage period did not significantly (*p* < 0.05) affect the evaluation of this aroma, which was almost identical to that of fresh pasta-filata cheeses. Overall, evaluation in terms of unacceptable aroma of LAB-enriched samples was positive and samples did not have any off-flavor marks. The sensory scoring of grassy, spicy and fruity aroma reported by the assessors’ panel was very low and did not change during the study (*p* < 0.05; 1.2 ± 0.4, 1.1 ± 0.3, and 1.2 ± 0.4, respectively). Aroma defects such as rancid, moldy and cowshed odor were observed mostly in cheese without the addition of LAB cultures, and significant differences (*p* < 0.05) between the RM and the CC were observed.

The addition of LAB during cheese manufacture substantially enhanced the taste of the pasta-filata cheeses. The values of desirable milky taste attribute of fresh cheeses were significantly (*p* < 0.05) higher in RM (4.2 ± 0.3) that in CC cheeses (3.0 ± 0.5). After 28 days of storage, a gradual decrease in milky taste was noticed for RM and CC cheeses (2.8 ± 0.4, and 2.2 ± 0.6, respectively). Descriptors associated with the taste of fresh cheese prevailed in LAB-enriched fresh cheeses (4.2 ± 0.7) in comparison with fresh CC (3.0 ± 0.8). Sensory evaluation of this attribute after the storage period significantly (*p* < 0.05) decreased for both the RM (2.8 ± 0.8) and CC (1.6 ± 0.7) groups of cheeses. Acidic taste is associated with the basic taste sensation for pasta-filata cheeses. The results of this descriptor ranged from 1.8 to 3.2, indicating a weak to moderate acidic taste. However, cheeses without LAB addition exhibited significantly (*p* < 0.05) higher scores (3.1 ± 0.9) for acidic taste than did RM cheeses (2.2 ± 0.8). Similarly, the perception of acidic taste suitability was evaluated and showed acceptability ranging from satisfactory to almost pleasant (3.9 ± 0.9). Furthermore, taste defects such as rotten, metallic, grassy, spicy and fruity defects were generally rated as unnoticeable (1.1 ± 0.4, 1.1 ± 0.4, 1.1 ± 0.3, 1.2 ± 0.5 and 1.1 ± 0.3, respectively). Several assessors also identified cowshed taste, but it was generally rated as unnoticeable (1.5 ± 0.6) in the RM group of cheeses in comparison with the CC group of cheeses (2.5 ± 0.9). Bitter taste was not affected by the adjunct culture used, but scores of the stored cheeses in the sensory acceptance test showed a tendency to increase approximately 0.4 points after 28 days of storage (1.4 ± 0.6 to 1.8 ± 0.8).

### Overall Acceptability

**FIGURE 5:**
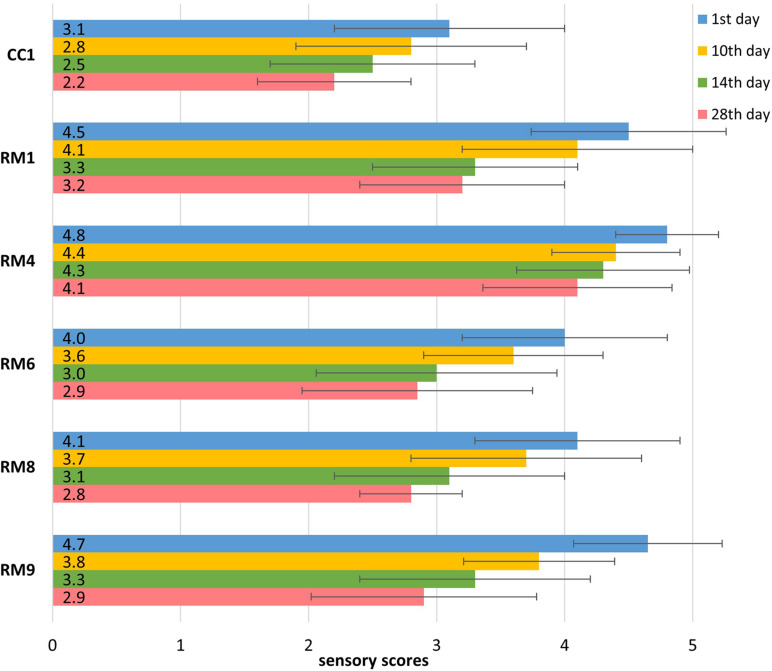
The qualitative sensory evaluation of overall acceptability of chosen pasta-filata cheese.

All of the abovementioned sensory aspects were assessed and expressed as the overall level of acceptability for the pasta-filata cheeses (Figure 5). The final evaluation of the overall quality has a key role in the total sensory consumer acceptance of prepared products. The mean values and standard deviations (SD) of the overall acceptability used in the quantitative descriptive analysis (QDA) for cheeses (1, 10, 14, and 28 days) are presented in Table 2. According to the overall preference ratings, CC1 tended to be the least preferred sample in terms of the overall quality regardless of storage period. Samples enriched with LAB were, on average, 37% more acceptable products than CC1. The highest average scores were obtained by RM4 and RM9 cheeses on the day of production (4.8 ± 0.4, and 4.7 ± 0.6, respectively). The storage period decreased the overall acceptability of the final products. However, 28-day-old RM cheeses were rated from 2.8 to 4.1, showing acceptability ranging from satisfactory to almost pleasant.

**TABLE 2:** Sensory scores of the overall acceptability of selected pasta-filata cheeses during storage period.

**Cheese**	**Inoculated cultures with intended final concentration**	**1st day**	**10th day**	**14th day**	**28th day**
CC1	none	3.1 ± 0.9^*a,y*^	2.8 ± 0.9^*a,y*^	2.5 ± 0.8^*a,y*^	2.2 ± 0.6^*a,z*^
RM1	Fresco 10^6^ CFU/ml	4.5 ± 0.8^*a,x*^	4.1 ± 0.9^*a,x*^	3.3 ± 0.8^*b,y*^	3.2 ± 0.8^*b,y*^
RM4	culture A 10^6^ CFU/ml	4.8 ± 0.4^*a,x*^	4.4 ± 0.5^*a,x*^	4.3 ± 0.7^*a,x*^	4.1 ± 0.7^*a,x*^
RM6	Fresco 10^6^ CFU/ml LA145 10^6^ CFU/ml	4.0 ± 0.8^*a,x*^	3.6 ± 0.7^*a,x*^	3.0 ± 0.9^*a,y*^	2.9 ± 0.9^*a,y*^
RM8	Fresco 10^6^ CFU/ml VT1 10^6^ CFU/ml	4.1 ± 0.8^*a,x*^	3.7 ± 0.9^*a,x*^	3.1 ± 0.9^*b,y*^	2.8 ± 0.4^*b,y*^
RM9	Fresco 10^6^ CFU/ml LGG 10^6^ CFU/ml	4.7 ± 0.6^*a,x*^	3.8 ± 0.6^*b,x*^	3.3 ± 0.9^*b,y*^	2.9 ± 0.9^*b,y*^

*1 – worst possible value, 5 – best possible value (results are mean values ± standard deviation of 10 evaluations). ^*a, b*^ – within a row, letters with a differing superscript are significantly different (*p* < 0.05). ^*x, y, z*^ – within a column, letter with differing superscript are significantly different (*p* < 0.05). LA145 – *Lb. acidophilus* LA145, VT1 – *Lcb. rhamnosus* VT1, GG – *Lcb. rhamnosus* GG.*

## Discussion

Traditional raw milk cheeses are of great importance to maintain national heritage and tend to display greater variability, and strong and unique sensory properties compared with cheeses from pasteurized milk (Pappa et al., 2019); however, the usage of raw milk carries a potential health risk (Verraes et al., 2015). Therefore, every effort should be made to minimize this risk, with the addition of LAB being one of the oldest and simplest options. This was also the aim of our study, to evaluate the effects of adjunct cultures not only on microbiological quality and safety but also on the sensory properties of final products.

Autochthonous LAB are necessary to transform curds into cheese (Settanni and Moschetti, 2010) and enhance the stability and sensory properties of the final products (Guarrasi et al., 2017; Todaro et al., 2018). The growth and fermentative metabolism of LAB, as a permanent component of raw milk microbiota, can act as a natural inhibitory barrier in a wide variety of fermented dairy products. The most effective inhibiting activity against pathogenic and spoilage microorganisms is achieved by the production of organic acids with a subsequent *pH* decrease. It is relevant not only during the curd production phase, in which release of soluble Ca and change in casein structure occurs, but also in later stages when lower *pH* results in lower hardness (Fuentes et al., 2015). In addition, the potential presence of bacteriocins, H_2_O_2_ and aromatic compounds limits the growth of undesirable microbiota, and LAB, as a strong competitor, reveal the competition for nutritional factors (nicotinamide, biotin or niacin, etc.) needed for the growth of the bacteria present (Marth and Steele, 2001; Østlie et al., 2003; McSweeney, 2004; Charlier et al., 2008; Lahtinen et al., 2011). In the case of small amounts or insufficient acid production, no or minimal inhibitory effects are observed, as we have previously shown for *E. coli* and *S. aureus* (Le Marc et al., 2009; Ačai et al., 2016; Valík et al., 2018). Therefore, the attempts of scientists and technologists are naturally focused on the best possible cultures that will have appropriate metabolic activity and, in addition, will contribute to the improvement of technological and sensory properties of products. In addition, the selected cultures have to maintain their viability to a sufficient degree during the entire production process, especially the heating steps and storage periods.

Guidone et al. (2015) and Reale et al. (2019) reported that the *Lactobacillus casei* group is the most frequently used adjunct in several cheese types. The *Lactobacillus casei* group is acid-resistant, tolerates stress factors encountered in food processing and in the gastrointestinal tract, has a low frequency of antibiotic resistant phenotypes and the absence of the most frequently acquired antibiotic resistance genes, is better adapted to the cheese environment and survives resolutely at high counts in different mature cheeses.

Regarding viability, the study by Cuffia et al. (2017) proved the viability of *Lcb. rhamnosus* GG at levels higher than 7.5 log CFU/g during the cheese-making process and during the whole storage period. The loss of viability at 62.5 ± 0.5°C for 10 min was only 0.44 ± 0.12 log CFU/g. Moreover, counts of total LAB were higher than 9.1 log CFU/g during 15 days of storage at 4°C regardless of the day of storage or *Lcb. rhamnosus* GG presence. Additionally, *Lb. acidophilus* LA5 survived the heating process in pasta-filata cheese production, as its counts decreased after 1 day of storage by only 0.5 log CFU/g, and during ripening, the counts remained stable above 8 log CFU/g (Cuffia et al., 2017). Additionally, in the study of Cuffia et al. (2019), the counts of *Lcb. rhamnosus* GG and *Lb. acidophilus* LA5 were higher than 8 log CFU/g, and the counts of *S. thermophilus* STI-14 were higher than 9 log CFU/g during 15 days of storage. Pappa et al. (2019) reported that *S. thermophilus*, as a thermotolerant bacterium, exhibits better viability during the process of heating acidified curds than do mixed mesophilic *L. lactis* starter strains. The heating process at 72°C for 2 min destroyed 91% (*S. thermophilus*, *Lc. lactis*, *Lb. casei*; actually less than 2 logs decrease) and 85% (*S. thermophilus*, *Lb. delbrueckii* ssp. *bulgaricus*) of selected starter LAB cultures; however, during 180 days of ripening and storage, the counts of LAB were above 7 log CFU/g. Moreover, they also reported that microbial recovery was much more pronounced in raw milk cheeses than in pasteurized milk cheeses. Additionally, in the study of Reale et al. (2019), the counts of the primary starter *S. thermophilus* were close to 9 log CFU/g, and during the steaming process they decreased by approximately 2 log CFU/g.

In this context, we used *Lcb. rhamnosus* GG, *Lcb. rhamnosus* VT1, and *Lb. acidophilus* and, as a starter culture, Fresco culture (consisting of *L. lactis* ssp. *lactis*, *L. lactis* ssp. *Cremoris*, and *S. salivarius* ssp. *thermophilus*) and culture A (consisting of *L. lactis* ssp. *lactis*, *L. lactis* ssp. *cremoris*, *Leuconostoc mesenteroides* ssp. *Cremoris*, and *Lb. acidophilus*) in pasta-filata cheese manufacture. During the steaming process, their counts also decreased to a similar degree as described above.

To minimize the risk and increase the technological and sensory properties of final products, it is also necessary for LAB to be the dominant microbes in pasta-filata cheeses at the end of the refrigerated storage period. This was the case for 224 pasta-filata cheeses prepared by Todaro et al. (2017), in which LAB dominated the microbial communities during the entire 180 days of storage at levels between 7-8 log CFU/g. In 192 cheeses presented in the work of Todaro et al. (2018), the counts of mesophilic cocci and bacilli were higher than 7.3 log CFU/g. Additionally, Minervini et al. (2012) observed that counts of mesophilic lactobacilli remained above 7.4 log CFU/g after 14 days of Fior di Latte (chemically acidified Italian pasta-filata) cheeses. In our case, in all 23 pasta-filata cheeses, after 28 days at 6 ± 0.5°C, the average counts of lactococci were 6.3 ± 1.2 log CFU/g, and the average counts of lactobacilli were 5.7 ± 1.6 log CFU/g. It might be a reason of either different composition of raw milk microbiota in regard to their metabolic activity, or development of non-cultivable non-starter LAB and their activity, and inhibitory effect of salting on starter culture LAB (in our case, the dominating bacteria in starter cultures were lactococci). Also the higher redox potential on the surface of “Nite” cheeses (their diameter is in average 0.5 cm) may not favor some LAB growth. In addition, in studies by Todaro et al. (2017, 2018) the cheeses were packed in 70% of N_2_ and 30% of CO_2_ atmosphere preferring the growth of anaerobic or facultative anaerobic LAB. Additionally, in the study by Šipošová et al. (2020) of 39 Slovak raw milk pasta-filata cheeses, the average counts of lactococci were 6.7 ± 1.1 log CFU/g; however, lower average counts of lactobacilli (4.4 ± 1.0 log CFU/g) were reported. Similarly, in the study by Tomáška et al. (2019), the levels of lactococci and lactobacilli were high, 6-8 log CFU/g in “Nite” cheeses from raw milk.

In addition, LAB do not always originate solely from starter culture, and their origin is also attributed to raw milk; therefore, in raw milk cheeses, their counts are naturally higher (Niro et al., 2014). This was also observed in our investigation, with lowest average counts of LAB in CC curds, followed by PM. The highest counts of LAB were observed in RM curds as a result of autochthonous LAB presence and addition of starter cultures. Fuentes et al. (2015) also reported that the addition of adjuncts significantly increased the counts of LAB after 30 days of ripening, in the case of lactobacilli by approximately 1–1.2 log CFU/g and in the case of lactococci by approximately 0.4–0.8 log CFU/g, depending on the adjuncts used, and their counts in 18 Oaxaca (Mexican non-aged pasta-filata) cheeses remained higher than 8.1 log CFU/g and 6.3 log CFU/g, respectively, for 24 days of storage at 8°C. Additionally, as reported by Niro et al. (2014), mesophilic bacteria showed an increasing trend during ripening and reached levels of approximately 7 log CFU/g from the initial 5-6 log CFU/g. In the case of total counts of LAB in 16 commercial Hispanic pasta-filata cheeses, their average value (6.8 log CFU/g) was similar to the abovementioned values; however, in 4 cheeses, there was less than 2.7 log CFU/g of total LAB (Jimenez-Maroto et al., 2016).

As Cuffia et al. (2019) showed, the addition of *Lcb. rhamnosus* GG or *Lb. acidophilus* LA5 during pasta-filata soft cheese manufacture did not influence their gross composition (moisture, fat, protein) or *pH* value, which remained stable during ripening. In our case, we were not able to determine the *pH* values of “Nite” cheeses, because the diameter of cheeses was less than that of the pH-meter sensor. In Slovak pasta-filata cheeses included in the study of Šipošová et al. (2020), the average *pH* of commercial samples was 5.36 ± 0.12 (data not published in the study), and the *pH* value of curds in this study (before steaming) averaged 5.32 ± 0.48. Moreover, based on studies by Minervini et al. (2012); Guidone et al. (2015), Cuffia et al. (2017; 2019) and Pappa et al. (2019), *pH* values of samples remained stable during the entire storage period and so the adjuncts did not contribute to the undesirable decrease of acids. Lower *pH* is frequently observed in cheeses produced with NSLAB adjuncts as a result of acetic acid production (Guidone et al., 2015).

Additionally, the final average *a*_*w*_ value (0.965 ± 0.006) of our 23 pasta-filata cheeses after 28 days of storage was consistent with values for cheeses in the study of Šipošová et al. (2020), where the average *a*_*w*_ value of 39 cheeses was 0.956 ± 0.012 (data not published in the study). In contrast, Todaro et al. (2017) reported higher *a*_*w*_ value (0.984) after 30 days and an *a*_*w*_ value of 0.971 after 180 days of storage at 4°C. Lower *a*_*w*_ values at the end of storage prevent the development of undesirable microbial populations (Robertson, 1993).

Considering the LAB inhibitory properties against the growth of CPS and *E. coli*, the most favorable effect was observed in cheeses with LAB as the adjunct. In the case of 24-h-old curds, the lowest counts of CPS and *E. coli* (2.4 log CFU/g for both) were observed in cheeses manufactured with culture A (in the case of CPS) or Fresco + *Lcb. rhamnosus* VT1. In contrast, CPS and *E. coli* counts in CC curds ranged from 3.60 – 5.82 log CFU/g and 5.30 – 6.72 log CFU/g, respectively. The steaming process naturally reduced microbial numbers; counts of CPS and *E. coli* in the CC group ranged from 3.88 – 4.40 log CFU/g and 3.92-4.78 log CFU/g, respectively. Fresh RM pasta-filata cheese with the lowest value of CPS (2.78 log CFU/g) was manufactured using the combination of Fresco culture and culture A, while the best fresh pasta-filata cheese with the lowest *E. coli* level (1.23 log CFU/g) was manufactured with the addition of Fresco culture and *Lb. acidophilus* LA145. At the end of storage at 6 ± 0.5°C, neither *E. coli* or CPS were detected in cheeses manufactured with the addition of Fresco culture combined with *Lcb. rhamnosus* GG, and *E. coli* was also not detected in cheese enriched with Fresco and A culture combined. Our results are consistent with the findings of Jimenez-Maroto et al. (2016); of 16 commercial Hispanic pasta-filata cheeses, none were found to be positive for the presence of CPS, and the counts of coliforms in 44% of the cheeses were lower than 10 CFU/g, and in 37% the counts were lower than 2 log CFU/g. In 3 cheeses, the counts were 3.2–4.1 log CFU/g. Additionally, in other studies, *E. coli* and CPS were either below the detection limit during the entire storage period (Todaro et al., 2017; 2018), were found at very low levels (Niro et al., 2014; Fuentes et al., 2015), or in Kashkaval cheeses, *E. coli* counts were below 10 CFU/g since day 12 (Pappa et al., 2019). Conversely, the level of CPS remained high in Kashkaval cheeses during the entire period, and in 30-day-old cheeses, it reached the highest value, 5.5 ± 0.4 log CFU/g. Similar results were observed in other Slovak raw milk commercial pasta-filata cheeses, where the average counts of *E. coli* and CPS were 1.85 ± 0.99 log CFU/g and 3.94 ± 0.99 log CFU/g, respectively (Šipošová et al., 2020). In raw milk “Nite” cheeses, the level of CPS was on the order of 2-3 log CFU/g. Higher counts of CPS may be related to post-steaming contamination, especially in manually prepared cheeses (Tomáška et al., 2019).

For the incorporation of LAB into food products, cultures used should be technologically suitable, such that they establish viability and efficacy throughout the storage. In addition, during refrigeration, adjunct strains should not lead to undesirable changes in texture, flavor, or aroma characteristics. The sensory attributes of cheeses, such as appearance and texture, are clearly visible, and thus are a prerequisite for consumer acceptance (Karimi et al., 2012).

The sensory profiles showed no noteworthy differences in appearance attributes between the LAB-enriched and control pasta-filata cheeses. Similarly, Cuffia et al. (2017), examining the acceptability of soft pasta-filata cheese (Fior di Latte type) enriched with *Lcb. rhamnosus* GG, found no differences in color from that of conventional commercial cheese (*p* < 0.05). Dinakar and Mistry (1994) also reported that some bifidobacteria had no significant effect on the appearance of cheddar cheese through 24 weeks of storage. Gomes et al. (2011) reported that cheese with LAB addition received lower scores for appearance. In contrast, Albenzio et al. (2013) and Braghieri et al. (2015) reported that incorporation of LAB enhanced the color uniformity of Scamorza cheese. The negative effect of storage period on the appearance of Slovak pasta-filata “Parenica” was also noted by Semjon et al. (2019). Fresh unsmoked samples showed a descriptor of appearance approximately 25% higher than for cheeses after a 14-day storage period. In our case, this attribute decreased approximately 12 – 36% (on average 22%) after 28 days of storage.

The type of adjunct culture in our study did not result in significant sensory differences in textural parameters. However, due to LAB incorporation, a higher appreciation of our sample texture was observed. Assessors evaluated that chewiness in LAB-enriched samples was enhanced. Cuffia et al. (2017; 2019) reported that the chewiness of probiotic pasta-filata cheese was comparable to that of the control cheese (non-probiotic). Conversely, Sandoval-Copado et al. (2016) showed that both Oaxaca cheese made with mesophilic (*L. lactis* ssp. *lactis* and *L. lactis* ssp. *cremoris*) and thermophilic (*S. salivarius* ssp. *thermophilus*) LAB cultures were less chewable than were cheeses from naturally acidified raw milk. In terms of elasticity, we observed positive changes and improvement of this mechanical textural attribute in cheeses with LAB. Addition of *Lb. acidophilus* LA5 and *Lcb. rhamnosus* GG has been previously reported to improve the elasticity of pasta-filata soft cheese (Cuffia et al., 2017, 2019). Nevertheless, Braghieri et al. (2015) reported that LAB incorporation into Scamorza induced lower elasticity. In our study, no significant differences were detected in elasticity after storage of pasta-filata cheeses; however, after storage, there was less tendency for the cheeses to return to its initial shape after being compressed. This trend was consistent with the findings of Kindstedt (1993), Fuentes et al. (2015) and Semjon et al. (2019). These authors have associated the modification of the elasticity of cheese due to LAB incorporation with proteolysis. Similarly, Reale et al. (2019) recorded the ranking for elasticity of Scamorza with LAB around the neutral point of the hedonic scale (5 points on a scale from 1 to 9) after 30 days of storage. In our study, we observed mean scoring just above the middle category (3.4 ± 0.6) at the end of the storage period. As was also observed in our study, Cuffia et al. (2019) reported no uniform effect of LAB addition into pasta-filata on mechanical attributes such as stickiness. Nevertheless, Albenzio et al. (2013) determined higher stickiness in LAB-enriched pasta-filata.

Many investigations have indicates that the addition of LAB has an impact on the aroma and taste of innovatively produced cheeses (Ryhänen et al., 2001; Ong et al., 2007; de Souza et al., 2008; Hoorde et al., 2010; Gomes et al., 2011; Guidone et al., 2015; Hammam et al., 2018). In our study, LAB-enriched pasta-filata cheeses received a better score for aroma intensity compared to the CC group. In contrast, some studies have concluded that scores for aroma intensity of pasta-filata cheese increased with adjunct culture (Minervini et al., 2012; Cuffia et al., 2017, 2019; Reale et al., 2019). Some of the aroma and taste descriptors described in our study are consistent with results obtained in previous artisanal pasta-filata cheese studies. Braghieri et al. (2015) and Albenzio et al. (2013) found that milk aroma in LAB-enriched cheeses was higher than in control samples. Sandoval-Copado et al. (2016) incorporated lactococci and streptococci into Oaxaca cheese and found that LAB-enriched cheeses featured lower intensity of undesirable cowshed aroma. Several studies focusing on the incorporation of LAB into pasta-filata have reported a higher level of sensory perception of bitterness (Sandoval-Copado et al., 2016; Cuffia et al., 2019; Reale et al., 2019) in comparison with conventional (non-LAB) cheeses. As also observed by Albenzio et al. (2013) and Sandoval-Copado et al. (2016), the scoring of acid taste was comparable with that of the control sample, but pasta-filata cheeses with the addition of LAB received slightly lower scores regarding intensity of acid taste. In contrast, Braghieri et al. (2015), Cuffia et al. (2017; 2019) and Reale et al. (2019) determined that the LAB addition in cheeses resulted in rejection by consumers with respect to higher acid taste in comparison to control cheese.

The sensory profile of pasta-filata cheeses showed that the incorporation of LAB enhanced the overall acceptability, and the most satisfactory overall acceptability after 28 days of storage at 6 ± 0.5°C was reached for cheese with the addition of culture A (RM4, Figure 5). Having better acceptability of LAB-enriched cheeses is consistent with findings of Minervini et al. (2012) and Braghieri et al. (2015), who reported that pasta-filata cheeses produced using adjunct LAB cultures induced higher values of an acceptability score than did with control cheeses. Cuffia et al. (2019), who inoculated pasta-filata with *Lb. acidophilus* LA5 and *Lcb. rhamnosus* GG, received similar or higher sensory scores for LAB-enriched samples compared to control (non-LAB-inoculated) samples. In the study performed by Reale et al. (2019), the sensory profiles of the control sample and LAB-enriched Scamorza cheese were comparable. All these results showed that LAB mostly improved the sensory properties of pasta-filata cheeses, as was also observed in our study, or at least did not reduce in consumer acceptance in comparison to the original product.

As expected, the overall acceptance of RM cheeses was negatively affected by storage time. Deterioration of cheeses was mainly attributed to the bitter taste and slight softness. Based on overall acceptability, RM products stored for 14 days received scores from the judges approximately 29% lower, and the appreciation of 28-day refrigerated cheeses decreased by 38% in comparison with fresh samples. Semjon et al. (2019) also noted a negative effect of 14-day storage period on the overall acceptability of unsmoked “Parenica” from 8.1 to 4.9. Similarly, Fuentes et al. (2015) noted that the acceptability of cheese for consumption decreased by approximately 36% after 16 days of storage in comparison with products immediately after preparation.

These results suggest that the addition of LAB can improve the sensory profile of pasta-filata cheese. The differences in perceived textural characteristics, aroma and taste depend on the metabolic activity of the strains used in the cheese-making process. Contribution to sensory properties is attributed to the higher accumulation of microbial metabolites in LAB-enriched cheeses. Generally, proteolysis plays a key role in the development of the typical sensory characteristics of a cheese (de Souza et al., 2008; Gomes et al., 2011; Karimi et al., 2012; Niro et al., 2014; Braghieri et al., 2015; Guidone et al., 2015; Hammam and Ahmed, 2019; Reale et al., 2019) with the production of different amino acids that are precursors for specific sensory active metabolites (see Introduction).

## Conclusion

In conclusion, beyond the fermentation activity of natural LAB microbiota, the use of starter cultures is strongly recommended in artisanal lump cheeses that use raw milk for pasta-filata cheese production. This is the only approach capable of assuring the initial dominance of LAB and supporting the growth of the natural LAB present in raw milk in competition with other undesirable bacteria. Moreover, the addition of dairy culture will also enhance the sensory acceptance of final products. At this point, we would like to emphasize the great importance of LAB, including their dairy cultures, in preserving the national gastronomic heritages that are registered and protected in the EU.

## Data Availability Statement

The raw data supporting the conclusions of this article will be made available by the authors, without undue reservation.

## Author Contributions

KK and IH carried out the experimental work. MK and AM analyzed the dataset. AM and ĹV set up the experimental design. AM, MK, and ĹV wrote the manuscript. All authors contributed to the article and approved the submitted version.

## Conflict of Interest

The authors declare that the research was conducted in the absence of any commercial or financial relationships that could be construed as a potential conflict of interest.
