# Supplementary figures and images for: Effect of Lactic Acid Bacteria Addition on the Microbiological Safety of Pasta-Filata Types of Cheeses
Source: Front Microbiol. 2020 Dec 7;11:612528. doi: 10.3389/fmicb.2020.612528 (PMC7750188; doi:10.3389/fmicb.2020.612528)

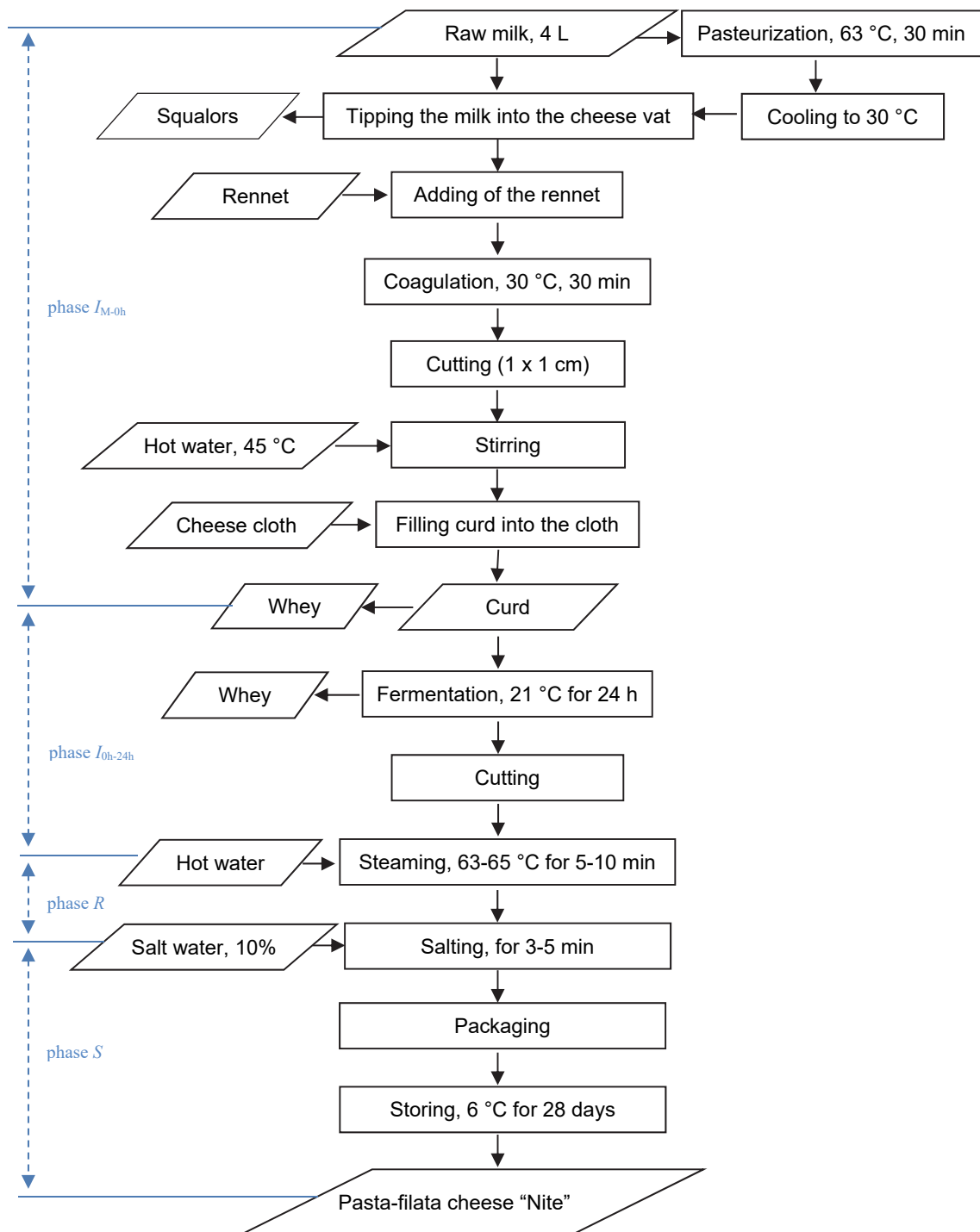

Supplement: Supplementary file 1 [file Data_Sheet_1.PDF]
